# Development and evaluation of an indirect ELISA using a multiepitope antigen for the diagnosis of intestinal schistosomiasis

**DOI:** 10.1017/S0031182023000409

**Published:** 2023-07

**Authors:** Karine Ferreira Lopes, Mariana Lourenço Freire, Dayane Costa Souza Lima, Martin Johannes Enk, Edward Oliveira, Stefan Michael Geiger

**Affiliations:** 1 Department of Parasitology, Institute of Biological Sciences, Federal University of Minas Gerais, Belo Horizonte, Minas Gerais, Brazil; 2 René Rachou Institute – Oswaldo Cruz Foundation, Belo Horizonte, Minas Gerais, Brazil; 3 Evandro Chagas Institute – Secretary of Health Vigilance, Ministry of Health, Ananindeua, Pará, Brazil

**Keywords:** Asparaginyl endopeptidase, cathepsin B, diagnosis, low parasitic load, multiepitope antigen, *Schistosoma mansoni*, serology

## Abstract

The laboratory diagnosis of intestinal schistosomiasis, carried out by detecting parasite eggs in feces, has low sensitivity when applied to individuals with low parasitic load. Serological tests can be more sensitive for the diagnosis of the disease. Therefore, the objective of this work was to develop and evaluate an ELISA-based immunoenzymatic assay, using a *Schistosoma mansoni* multiepitope antigen (ELISA IgG anti-SmME). For this, the amino acid sequences of *S. mansoni* cathepsin B and asparaginyl endopeptidase were submitted to the prediction of B cell epitopes and, together with peptide sequences obtained from earlier works, were used in the construction of a minigene. The multiepitope protein was expressed in *Escherichia coli* and the performance of the ELISA IgG anti-SmME for schistosomiasis was evaluated using serum samples from 107 individuals either egg positive or negative. In addition, 11 samples from individuals with other helminth infections were included. The ELISA IgG anti-SmME showed a sensitivity of 81.1% and a specificity of 46.1%. Further analysis revealed a 77.2% sensitivity in diagnosis of individuals with egg counts of ≤12 epg (eggs per gram feces) and 87.5% for individuals with 13–99 epg. It is worth mentioning that, to our knowledge, this was the first study using a multiepitope recombinant antigen in an ELISA for diagnosis of intestinal schistosomiasis, which demonstrated promising results in the diagnosis of individuals with low parasitic loads.

## Graphical abstract

**Figure:**
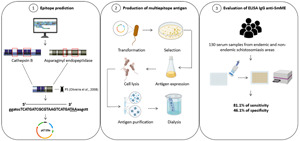

## Introduction

Schistosomiasis is a neglected tropical disease (NTD) caused by parasite flatworms of the genus *Schistosoma*. The disease is still considered a major public health problem worldwide, especially in impoverished populations (Gryseels *et al*., 2006; Colley *et al*., 2014; McManus *et al*., 2018). Approximately 240 million individuals are estimated to be infected, mainly by species *Schistosoma haematobium*, *Schistosoma japonicum* and *Schistosoma mansoni* (Weerakoon *et al*., 2015). Moreover, the disease is distributed over 78 countries in tropical and subtropical regions and more than 700 million individuals live in endemic areas and are at risk of infection (World Health Organization, 2021). In Brazil, schistosomiasis is solely caused by *S. mansoni*, and the latest national prevalence survey from 2010 to 2015 resulted in estimates of a positivity rate of around 1.0% (Katz, 2018).

Usually, laboratory diagnosis relies on the detection of schistosome eggs in stool samples, e.g. by using the Kato–Katz thick-smear technique (Katz *et al*., 1972). The technique is cheap, easy to perform and of high sensitivity when applied in individuals with a medium-to-high parasitic load, or when additional stool samples are collected in consecutive days and an increased number of slides are analysed (Enk *et al*., 2008; Speich *et al*., 2010; Siqueira *et al*., 2011; PAHO, 2014; Oliveira *et al*., 2018). However, its sensitivity significantly decreased when used for diagnosis in individuals with a previous history of praziquantel (PZQ) treatment, or in areas of low prevalence, where most of the infected individuals had a low parasitic load (de Vlas and Gryseels, 1992). Therefore, and within the scope of the new millennium goals for the control of the most common NTDs (Horwitz, 2018), it is necessary to develop more sensitive and specific techniques for schistosomiasis diagnosis. This holds true especially in areas of low endemicity, for individual case detection, or during epidemiological surveillance and screening of individuals in order to provide treatment (Gomes *et al*., 2014).

In the definitive mammalian host, *S. mansoni* secretes and excretes a wide variety of different soluble products (Hokke *et al*., 2007). These antigens induce cellular and humoral immune responses, and the antigen-specific antibodies can be measured using serological tests (Planchart *et al*., 2007). The diagnostic potential of some selected antigens was evaluated in various studies (Rotmans *et al*., 1981; Kanamura *et al*., 1998; Coulibaly *et al*., 2013). Ruppel *et al*. (1985*a*) demonstrated that proteins with a molecular weight of 31 kDa (Sm31) and 32 kDa (Sm32) were detected by murine antibodies after 4 weeks of experimental infection and these same antigens were recognized by serum samples from humans with intestinal schistosomiasis (Ruppel *et al*., 1985*b*). Based on the nucleotide and amino acid sequences, Sm31 was characterized as a cathepsin B, with proteolytic activity (Klinkert *et al*., 1989) and Sm32 was classified as a cysteine protease (Ishii, 1994). While Sm31 is directly involved in the digestion of haemoglobin in the parasite digestive tube (Brindley *et al*., 1997), Sm32 is involved in the proteolytic activation of other enzymes that perform this function (Dalton *et al*., 1996). In earlier works, cathepsin B from *S. mansoni* was expressed as a fusion protein with the amino-terminal region of the RNA replicase of the phage MS2 in *Escherichia coli*. However, the fusion protein aggregated in the cytoplasm and could only be solubilized under intense denaturing conditions (Klinkert *et al*., 1988). Furthermore, this same protein was expressed in insect cells, but the yield obtained was extremely low (Götz *et al*., 1992).

In experiments with clones of an adult worm cDNA library, the clone designated as ET03 was the most reactive during screening with anti-Sm31/Sm32 antibodies eluted from nitrocellulose strips. Sequencing of this clone provided a 682 nucleotide sequence and the translation of the cDNA resulted in only 1 open reading frame (ORF), corresponding to 57 amino acids (Valli *et al*., 2007). In 2006, Oliveira *et al*. (2006) synthesized 7 peptides, of which 3 were based on the amino acid sequence of cathepsin B and 2 on the ORF sequence of clone ET03. When used in an enzyme-linked immunosorbent assay (ELISA), these peptides showed high immunoreactivity against a serum pool from individuals with intestinal schistosomiasis. Then, this same group standardized an immunoassay (ELISA-Pp), using a mixture of these 5 synthetic peptides and the test showed a sensitivity and specificity of 86.8 and 94.2%, respectively, which emphasized the potential of the multiple epitopes antigen cocktail (Oliveira *et al*., 2008). Based on these earlier studies, the objective of the present work was to develop and evaluate an ELISA for the diagnosis of intestinal schistosomiasis, using a multiepitope antigen, composed of *S. mansoni* cathepsin B and asparaginyl endopeptidase epitopes, in combination with sequences of the previously evaluated peptides (Oliveira *et al*., 2008) and produced by recombinant DNA technology.

## Materials and methods

### Multiepitope antigen production

#### Multiepitope gene construction

The amino acid sequences of cathepsin B (access number M21309) and asparaginyl endopeptidase (access number AJ250582.1) were obtained through GenBank access (http://www.ncbi.nlm.nih.gov/Genbank/index.html) and subjected to epitope prediction by the BepiPred 2.0 predictor (Jespersen *et al*., 2017) and the method described by Kolaskar and Tongaonkar (1990), to identify potentially antigenic peptides. The regions with a high antigenicity score were defined and 4 peptide regions, named P1, P2, P3 and P4 were selected (Supplementary Figs S1 and S2). In addition, peptides from the cathepsin B amino acid sequence, published by Noya *et al*. (2001), and the ORF of the clone ET03, by Valli *et al*. (2007), previously evaluated by Oliveira *et al*. (2008), were included and designated P5 (Table 1). The selected peptides were back-translated to their respective nucleotide sequences, using BioEdit 7.2 software and sent to GenScript (Piscataway, NJ, USA), where P1–P5 antigen were optimized, including cleavage sites for *Bam*HI (5′), *Hind*III (3′) and stop codon (3′). Next, the multiepitope sequence was synthesized, and inserted between the *Bam*HI and *Hind*III restriction enzyme sites of plasmid pET-28a (+). The construction was named pET28a-P1-P5.

**Table 1.** Peptide sequences contained in the multiepitope antigen (P1–P5)

Peptide	Origin protein	Amino acid sequence
P1	Cathepsin B	99-WPGCKSIATIRDQSRCGSCWSFGAVEAMSDRSCIQSGGKQNVELSAVDLL TCCESCGLGCEG-160
P2	Cathepsin B	215-RCKQTCQRKYKTPYTQDKHRGKSSYNVKNDEKAIQKEIMKYGPVEASFT VYEDFLNYKSGIYK-277
P3	Asparaginyl endopeptidase	198-FQQILPSNLSIYATTAANPTECSYSTFCGDPTITTCLADLYSYNWIVDSQTH HLTQ-253
P4	Asparaginyl endopeptidase	375-QTLDCTESVYEQFKSKCFTLQQAPEVGGHFSTLYNYCAD-413
P5	Cathepsin B	81-DHNDWNVEIPSNFDSRKKWP-100
	Cathepsin B	221-QRKYKTPYTQDKHRGKSSYN-240
	Cathepsin B	241-VKNDEKAIQKEIMKYGPVEA-260
	ORF of clone ET03	5-HRQFCETKLIVLCRIFSNTH-24
	ORF of clone ET03	34-TVESLAIIILLKCAQVCIKI-53

#### Expression of multiepitope antigen

The pET28a-P1-P5 construction was used to transform *E. coli* BL21 pLysS competent cells by thermal shock (15 min on ice, 1 min at 42 °C and 10 min on ice). The cells were plated on Luria–Bertani agar containing 30 *μ*g mL^−1^ kanamycin (Sigma-Aldrich, St. Louis, MO, USA) and cultivated in an incubator overnight, at 37 °C. The selected clones were tested for the presence of P1–P5 genes through the restriction analysis with *Bam*HI and *Hind*III enzymes. Transformed colonies were cultured in 1 L of terrific broth medium, containing 30 *μ*g mL^−1^ of kanamycin in a shaker at 37 °C and 200 rpm. During the logarithmic growth phase [optical density (OD_590_) = 0.6–0.8], the recombinant protein expression was induced by 1 mm IPTG (isopropyl-*β*-d-thiogalactopyranoside). The cells were cultivated at 37 °C in a shaker for 4 h at 200 rpm, according to previously defined conditions. To purify the protein, 1 L bacterial culture was centrifuged at 4800 ***g*** for 30 min. The harvested cells were lysed for protein purification under denaturing conditions as previously described (Tran *et al*., 2005; Ntumngia and Adams, 2012), with some modifications. Briefly, the bacterial pellet was resuspended in lysis buffer (50 mm Tris buffer, pH 8.0, 500 mm NaCl, 0.2 mm EDTA, 3% sucrose, 1% Triton X-100, 200 *μ*g mL^−1^ lysozyme, 1 mm phenylmethylsulphonyl fluoride and 20 *μ*g mL^−1^ DNAse) and the cell suspension was sonicated on ice in an ultrasonic processor (VC-750, Sonics Vibra-Cell) by 5× 30 s pulses at 40% intensity. Cell debris was removed by centrifugation at 13 000 ***g*** for 30 min at 4 °C and inclusion bodies were washed twice (50 mm Tris buffer, 3 m urea, EDTA 0.2 mm, 500 mm NaCl, pH 8.0), sonicated and centrifuged. The solubility of P1–P5 antigen was evaluated by sodium dodecyl sulphate-polyacrylamide gel electrophoresis (SDS-PAGE).

#### SDS-PAGE and Western blotting

To confirm the expression and analyse the solubility of P1–P5 antigen, basic SDS-PAGE was performed with subsequent Western blotting according to Laemmli (1970) and Towbin *et al*. (1979), respectively. The proteins and molecular weight markers (Precision Plus Protein Dual Color Standards, Bio-Rad Laboratories, Hercules, CA, USA; Amersham ECL Rainbow Markers, GE Healthcare, Chicago, IL, USA) were separated on the 12% SDS-PAGE gel, and transferred onto a nitrocellulose membrane (Amersham Protran, 0.45 *μ*m – GE Healthcare, Chicago, IL, USA) at 300 mA for 2 h at 4 °C in transfer buffer (25 mm Tris, 192 mm glycine, 20% methanol). After that, the membrane blocking was performed with Tris-buffered saline (TBS) (250 mm Tris, 150 mm NaCl, 3 mm KCl, pH 7.4), containing 0.05% Tween 20 and 5% non-fatty dry milk (TBS-T-Milk 5%), for 2 h at room temperature. The membrane was washed thrice with washing buffer (TBS containing 0.05% Tween 20) and incubated with monoclonal 6x-His-tag antibody conjugated to peroxidase (Thermo Fisher Scientific, Waltham, MA, USA), diluted to 1:3000 in TBS-T-Milk 1%. Following 3 washes, the membrane was incubated with a secondary anti-mouse immunoglobulin G (IgG) antibody, coupled to peroxidase (Sigma-Aldrich), diluted to 1:30 000 for 1 h at room temperature. To visualize the recognized antigen–antibody complexes, the membrane was incubated with ECL™ Prime Western Blotting solution (GE Healthcare UK Ltd., Buckinghamshire, UK) and the results were captured using ImageQuant LAS 4000 (GE Healthcare, Chicago, IL, USA).

#### Purification of multiepitope antigen

The inclusion bodies were solubilized (6 m GuHCl, 0.2 m NaCl, 11.5 mm NaH_2_PO_4_, 10 mm Tris, 10 mm BetOH, pH 7.8) and harvested by centrifugation at 10 900 ***g*** for 30 min at 4 °C. The pellet was resuspended in buffer solubilization and incubated with Ni Sepharose high-performance resin (GE Healthcare Life Sciences, Uppsala, Sweden) for 1 h at 4 °C. The resin was collected by centrifugation at 1360 ***g*** for 5 min and the recombinant protein was purified in Econo-Pac^®^ chromatography columns (Bio-Rad Laboratories), according to the manufacturer's instructions, using elution buffer (10 mm phosphate buffer, 8 m urea, 10 mm imidazole, 10 mm BetOH, pH 4.3). Purified P1–P5 antigen were subjected to successive dialysis against elution buffer, containing 6 m, 4 m and 2 m urea for 3 h in each buffer at 4 °C. Then, the antigen was dialysed in a dialysis sack (molecular weight cut-off 12 000 Da, Sigma-Aldrich) against phosphate-buffered saline (PBS, 2.1 mm NaH_2_PO_4_, 8.3 mm Na_2_HPO_4_, 0.15 m NaCl, pH 7.3), also for 3 h, at 4 °C. Lastly, P1–P5 antigen were recovered, and the protein concentration was determined by the colorimetric bicinchoninic acid method (Thermo Fisher Scientific). The multiepitope antigen (P1–P5) was used to standardize an enzyme-linked immunoassay, named ELISA IgG anti-SmME (ELISA-based immunoenzymatic test, using a *Schistosoma mansoni* multiepitope antigen).

### Performance evaluation of the ELISA IgG anti-SmME

#### Serum samples

**Figure 1.**
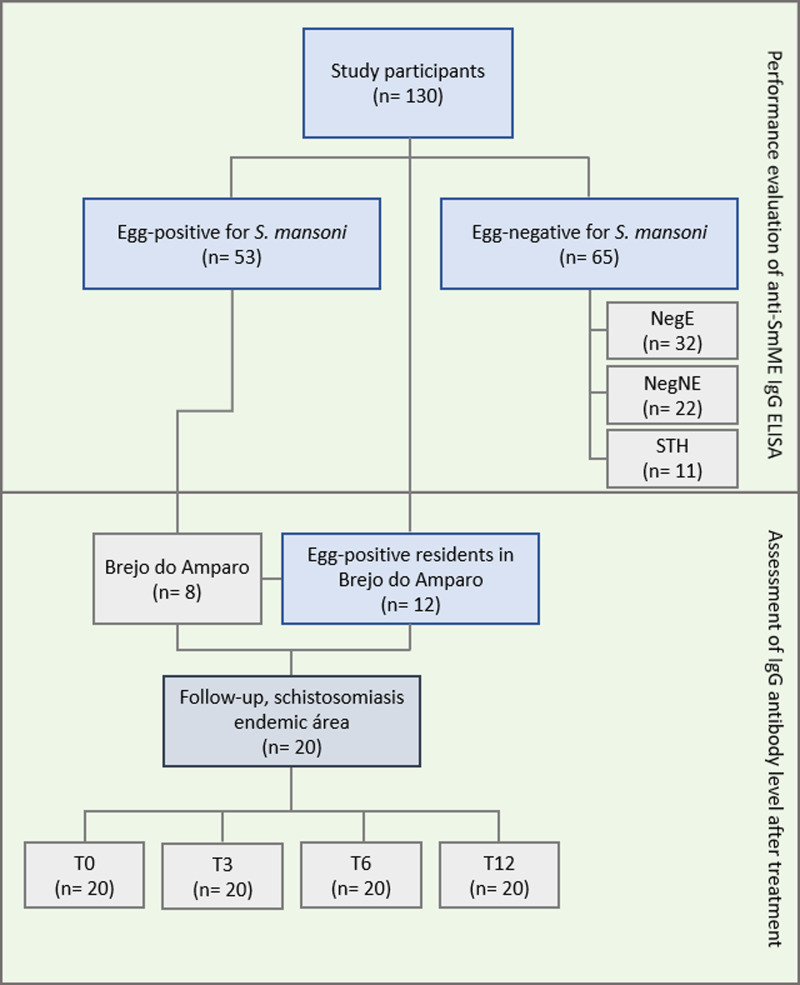
Flowchart for infection groups and number of serum samples used in the ELISA IgG anti-SmME. In the first phase, the assay performance was evaluated and carried out with a group of 118 serum samples, 53 belonging to the egg-positive (POS) group and 65 from the egg-negative group, according to parasitological techniques. In addition, individuals from the negative group were subdivided into egg-negatives from endemic areas (NegE), egg-negatives from non-endemic areas (NegNE) and egg-negatives infected with soil-transmitted helminths (STH). The second phase consisted of assessing the level of IgG antibodies after PZQ treatment. For this, samples of 12 individuals residing in Brejo do Amparo, plus a subgroup of 8 individuals included in the first phase, totalling 20 individuals, were subjected to the subsequent tests. These individuals participated in the follow-up study at 3, 6 and 12 months post-treatment.

Samples were chosen from the serum collection of the Laboratory of Intestinal Helminthiasis, located at the Institute of Biological Sciences of the Federal University of Minas Gerais properly stored in an ultra-freezer at −80 °C. The samples were collected during epidemiological studies on intestinal schistosomiasis (Oliveira *et al*., 2018; Sousa *et al*., 2019) and are covered by approvals from the National Brazilian Ethics Committee on Research with Human Subjects (DECIT project CAAE no. 21824513.9.0000.5091 for the areas Brejo do Amparo-MG and Primavera-PA, CAAE no. 55620116.2.0000.5149 for sera from the Central Region of Minas Gerais and CAAE no. 47200115.8.0000.5149 for Gameleira, Bom Jantar and Pindaibal). Serum samples from 130 residents of 4 different locations were used (Fig. 1). Participants were between 6 and 68 years of age and 55 were female and 75 were male. To determine the sensitivity of the ELISA, serum samples from 53 participants with intestinal schistosomiasis (POS group) were used. The group was composed by individuals living in the following localities: 2 rural communities localized in the municipality of Januária (*n* = 39), northern region of Minas Gerais, Brazil; municipality of Jaboticatubas (*n* = 8), central region of Minas Gerais, Brazil and municipality of Primavera (*n* = 6), located in the north-eastern region of Pará, Brazil.

The specificity of the ELISA was evaluated in the control group, composed by 65 serum samples of *S. mansoni*-negative individuals. The samples were distributed into the following subgroups:

*Negatives of endemic area* (NegE): composed by 32 serum samples of healthy individuals living in Jaboticatubas (*n* = 12), central region of Minas Gerais, classified as a municipality of low endemicity, as the prevalence of the disease resulted in less than 5% (unpublished data); and Bom Jantar (*n* = 10) and Gameleira (*n* = 10), rural communities localized in the municipality of Januária, northern Minas Gerais, formerly endemic areas for intestinal schistosomiasis. Bom Jantar and Gameleira do not have recent data available from the Schistosomiasis Control Program Information System (SISPCE), but there were positive cases reported by the local health authorities during past examinations.

*Negatives from non-endemic areas* (NegNE): composed by 22 serum samples from healthy individuals living in Morro do Pilar (*n* = 20), municipality in the central region of Minas Gerais; and Primavera, Pará (*n* = 2). Currently there are no data, and no cases were reported in the database of the SISPCE for the municipality of Morro do Pilar. In the municipality of Primavera, the transmission of schistosomiasis is focal, however, geohelminth infections were frequent.

*Infection group with soil-transmitted helminths* (STH): composed of 11 serum samples from individuals living in Primavera, infected with hookworm (*n* = 8), *Ascaris lumbricoides* (*n* = 1), hookworm and *A. lumbricoides* co-infection (*n* = 1) and *Trichuris trichiura* (*n* = 1).

The positive diagnosis of intestinal schistosomiasis or other helminthiases was confirmed by the Kato–Katz technique (Katz *et al*., 1972) using at least 2 slides prepared from 1 fecal sample. Additionally, fecal samples from individuals from northern Minas Gerais were characterized by Helmintex (Teixeira *et al*., 2007) and saline gradient (Coelho *et al*., 2009) techniques, as outlined elsewhere (Oliveira *et al*., 2018). Also, a subgroup of 20 *S. mansoni*-infected individuals who were diagnosed by the same parasitological techniques and by real-time polymerase chain reaction technique was analysed, as previously described (Magalhães *et al*., 2020). These individuals were egg-positive for *S. mansoni* at the beginning of the collection of fecal samples and, after treatment with PZQ, they remained egg-negative at 3, 6 and 12 months post-treatment and were included in order to evaluate the kinetics of antigen-specific IgG antibody levels up to 1-year post-treatment and parasitological cure.

### Enzyme-linked immunosorbent assay

Based on previous checkerboard titration analysis, the ELISA using the recombinant P1–P5 antigen, named ELISA IgG anti-SmME, was performed as follows: polystyrene microplates (Costar 3590, Corning Incorporated, Corning, NY, USA) were coated with 50 *μ*L per well of rP1-P5 diluted to 0.5 *μ*g mL^−1^ in carbonate bicarbonate buffer (10 mm Na_2_CO_3_, 30 mm NaHCO_3_, pH 9.6) and incubated for 30 min in a humid chamber at 37 °C, followed by further overnight incubation at 4 °C. The microplates were washed 5 times with PBS containing 0.05% Tween 20 (PBS-T) and incubated with 200 *μ*L per well of 5% non-fatty milk diluted in PBS-T (PBS-T-Milk 5%) for 2 h in a humidified chamber at 37 °C. After several fresh washings, 50 *μ*L of human serum samples diluted to 1:100 in PBS-T-Milk 1%, were added in duplicates into the wells and the microplates were incubated for 1 h in a humidified chamber at 37 °C. The microplates were washed again and incubated with secondary anti-human IgG antibody, coupled to peroxidase (Sigma-Aldrich) diluted to 1:30 000 in PBS-T-Milk 1%, for 1 h in a humidified chamber at 37 °C. Finally, the microplates were washed (5×), and the antigen–antibody interaction was revealed by addition of 50 *μ*L of a substrate solution (tetramethylbenzidine and hydrogen peroxide, Sigma-Aldrich). The microplates were incubated for 20 min at room temperature in the dark, and the enzymatic reaction was stopped with the addition of 50 *μ*L of 2 N sulphuric acid. The microplates were read at 450 nm in an automated microplate ELISA reader (VersaMax Microplate Reader, Molecular Devices, San Jose, CA, USA). Samples with absorbance readings above the cut-off, defined by the analysis of the receiver operating characteristic (ROC) curve, were considered positives.

### Statistical analysis

The database was built in Microsoft Office Excel 2010 spreadsheets and analysed using GraphPad Prism, version 8.0 (San Diego, CA, USA) or Open-Epi online, version 3.01 (Dean *et al*., 2013). The normal distribution of data was verified by the Shapiro–Wilk normality test. The ROC curve was used to determine the cut-off point using 53 participants with intestinal schistosomiasis (POS group) and 33 serum samples from *S. mansoni*-negative individuals belonging to the NegNE and STH groups. A comparison of the mean absorbance readings of 2 groups was performed using the Mann–Whitney *U* test, while the comparison of the 4 groups was performed using the Kruskal–Wallis test. Within the groups of participants before and after treatment, the comparison was performed using the Friedman's paired test. The OD values of the positive scores were compared with the parasitic load, using Spearman's non-parametric correlation test. A significance level of *P* ⩽ 0.05 was adopted for all statistical tests. The program Open-Epi was used for the calculation and analysis of serological parameters, such as sensitivity, specificity, positive (PPV) and negative-predictive values (NPV), accuracy and kappa index of ELISA results. The relationship between kappa index values and the degree of concordance was interpreted according to the scale of Landis and Koch (1977).

## Results

### Composition and analysis of P1–P5 gene

After the prediction of B cell epitopes, 2 peptide sequences were selected from cathepsin B, named P1 and P2, and 2 from asparaginyl endopeptidase, P3 and P4, which showed high antigenic indices. These peptides, together with the P5 sequence, previously used in the ELISA-Pp (Oliveira *et al*., 2008), were back-translated and used in the construction of the minigene coding for the multiepitope antigen (P1–P5 antigen). The gene contained 960 nucleotide base-pairs, which encodes a protein with 320 amino acids, with a theoretical molecular weight of 36.4 kDa and an isoelectric point of 8.29.

### Production of the recombinant P1–P5 antigen

**Figure 2.**
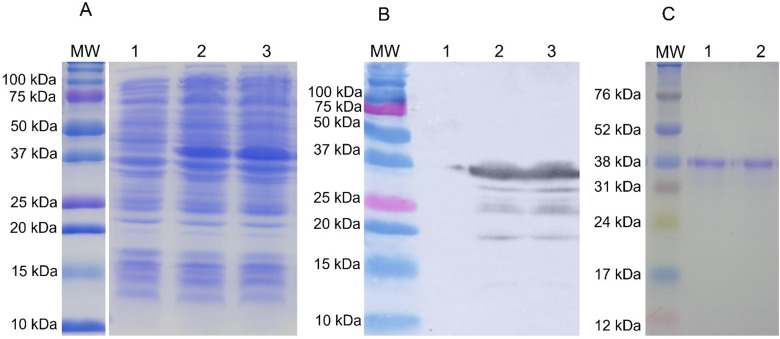
Production of recombinant P1–P5 antigen. (A) 12% SDS-PAGE with culture lysate before (lane 1) and 4 h after induction with IPTG (lanes 2–3). (B) Western blotting using monoclonal 6x-His tag antibody against recombinant P1–P5 antigen. (C) 12% SDS-PAGE with purified recombinant P1–P5 antigen (lanes 1–2). MW: molecular weight marker [(A) and (B): Precision Plus Protein Dual Color Standards, Bio-Rad Laboratories; (C): Amersham ECL Rainbow Markers, GE Healthcare, Chicago, IL, USA].

The construct pET28a-P1-P5 was used to transform *E. coli* BL21 pLysS and the recombinant protein was expressed after addition of 1 mm IPTG. SDS-PAGE analysis showed the expression of P1–P5 at different times after induction, with maximized expression after 4 h of incubation in a shaker at 37 °C and 200 rpm (Fig. 2A). The confirmation of expression of P1–P5 recombinant proteins was performed by western blotting, through visualization of a highly expressed approximately 38 kDa band, immunoreactive to a 6x-His-tag antibody (Fig. 2B). After large-scale expression, the solubility test indicated that the recombinant protein was found mainly in the insoluble fraction of the cell lysate. Figure 1C shows purified P1–P5 proteins at the expected molecular weight (lanes 1 and 2), with a concentration calculated to 410 *μ*g mL^−1^ by the Bradford assay.

### ELISA IgG anti-SmME for the detection of antigen-specific IgG antibodies in patients with intestinal schistosomiasis

**Figure 3.**
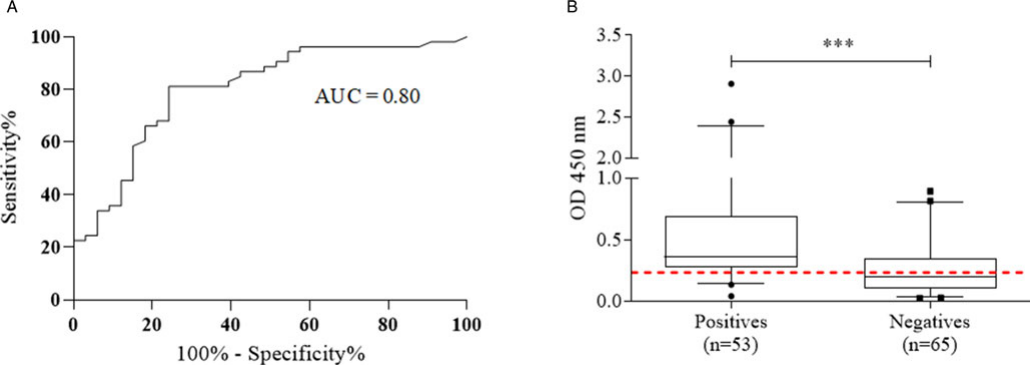
Evaluation of the ELISA IgG anti-SmME for the serological diagnosis of intestinal schistosomiasis. (A) Analysis of the ROC curve of the ELISA IgG anti-SmME, performed with sera from 53 egg-positive samples (POS) group and from 33 *Schistosoma mansoni* egg-negative individuals from non-endemic areas (NegNE) or with other 11 confirmed soil-transmitted helminth infections (STH). (B) Overall IgG antibody immunoreactivity for the ELISA IgG anti-SmME in the groups of egg-positive and egg-negative participants for intestinal schistosomiasis. The graph was constructed from the mean OD readings of 53 serum samples of egg-positive individuals and 65 egg-negative individuals from endemic (*n* = 32) and non-endemic areas (*n* = 22) and from individuals infected with other helminth infections (*n* = 11). The bars indicate median values, the boxes the 25 and 75% intervals and the whiskers the 5 and 95 percentiles. The red dotted line indicates the calculated cut-off point, according to ROC curve analysis (OD = 0.235). ***P < 0.001.

**Figure 4.**
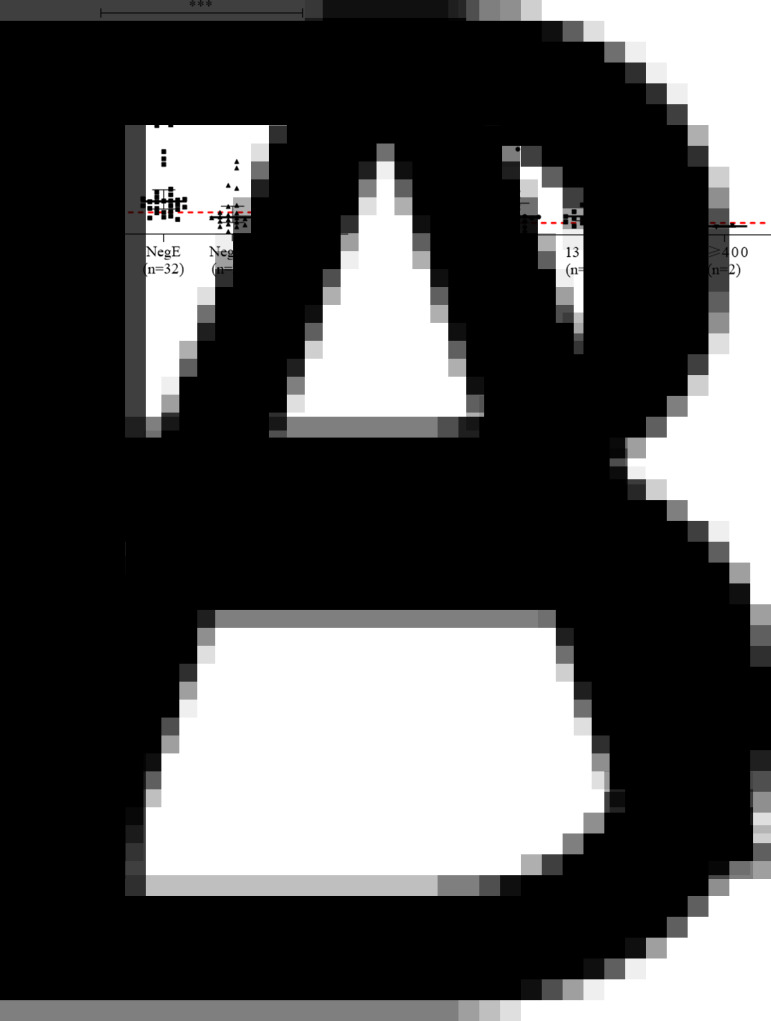
Individual and mean IgG antibody immunoreactivity (OD) against P1–P5 antigen in egg-positive participants and subgroups of egg-negative participants. (A) IgG immunoreactivity of antibodies against P1–P5 antigen in egg-positive individuals (POS), individuals from endemic (NegE) and non-endemic areas (NegNE) and in individuals with soil-transmitted helminth infections (STH). The graph was constructed by average of the absorbance readings of 53 *S. mansoni* egg-positive and 65 *S. mansoni* egg-negative samples. (B) Individual and mean IgG antibody immunoreactivity in *S. mansoni* egg-positive participants stratified by parasitic load. Individual *S. mansoni* parasitic loads expressed as mean epg values considered as very low (⩽12), low (13–99), medium (100–399) and high (⩾400). The number of epg was calculated from 2 Kato–Katz slides of 1 fecal sample. Fecal samples classified as egg negative in 2 Kato–Katz slides, but positive for *S. mansoni* eggs in other parasitological techniques, were classified as epg <12. Error bars indicate the median and interquartile ranges of 25 and 75% and the red dotted line the cut-off point (0.235). **P < 0.01, ***P < 0.001.

The performance of the ELISA IgG anti-SmME in detecting individuals with intestinal schistosomiasis was evaluated in a group of 118 serum samples. Analysis of the area under the curve (AUC) using the ROC curve demonstrated a discrimination power between individuals with and without intestinal schistosomiasis of 0.80 (AUC = 0.80) (Fig. 3A). A cut-off of 0.235 was defined based on the best accuracy (61.8%) and there was a significant discrimination between egg-positive and egg-negative groups (*P* < 0.0001) (Fig. 3B). The sensitivity of ELISA IgG anti-SmME was 81.1% and specificity was 46.1%, with PPV and NPV of 55.1 and 75.0%, respectively. Finally, the agreement between ELISA IgG anti-SmME and the reference test (2 KK slides and/or Helmintex^®^ and saline gradient) showed only fair concordance (*κ* = 0.26) (Table 2). The comparison of OD values between sera from egg-positive individuals and egg-negative individuals resident in non-endemic areas revealed a significantly higher immunoreactivity against P1–P5 antigen in infected participants (*P* = 0.0013) and when compared with individuals with other helminth infections (*P* = 0.0001). In addition, egg-negative individuals from endemic areas had a significantly elevated serum immunoreactivity than individuals from non-endemic areas (*P* = 0.01) and sera from individuals infected with other helminth species (*P* = 0.0007) (Fig. 4A). The ELISA IgG anti-SmME resulted in a good performance for the diagnosis of low-intensity infections, e.g. it was able to identify 17 out of 22 (77.2%) individuals with ⩽12 epg, 21 out of 24 individuals (87.5%) with 13–99 epg and 100% of individuals with 100–399 epg (Fig. 4B). Finally, no significant correlation was obtained between IgG antibody levels (OD) and individual *S. mansoni* egg counts (epg) (*r* = 0.009).

**Table 2.** Performance of the ELISA IgG anti-SmME in comparison with parasitological results from a composite reference test (2 Kato–Katz slides of a fecal sample or by the Helmintex® and saline gradient techniques) to classify participants into *Schistosoma mansoni*-infected (*n* = 53) and egg-negative individuals (*n* = 65)

Cut-off	Sens. (%) [95% CI]	Spec. (%) [95% CI]	PPV (%) [95% CI]	NPV (%) [95% CI]	Accuracy (%) [95% CI]	Kappa [95% CI]
0.301	81.1 [68.64–89.41]	46.1 [34.59–58.15]	55.1 [44.11–65.67]	75.0 [59.81–85.81)	61.8 [52.86–70.12]	0.26 [0.09–0.42]

95% CI, 95% confidence interval; PPV, positive-predictive value; NPV, negative-predictive value.

### Evaluation of IgG antibody levels after PZQ treatment

**Figure 5.**
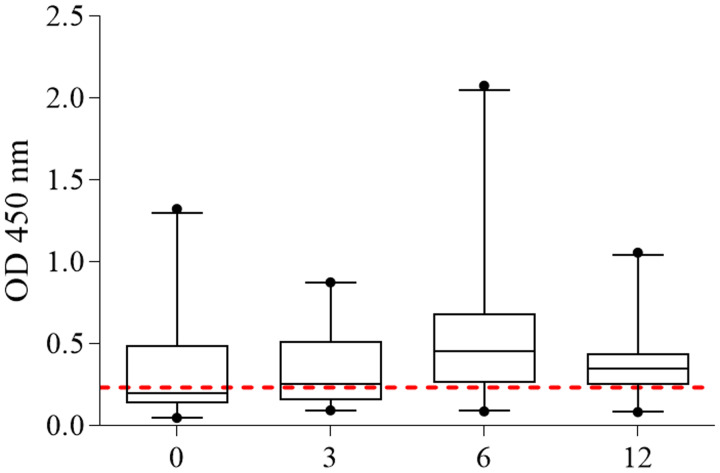
Kinetics of the IgG antibody measured by ELISA IgG anti-SmME in a group of *S. mansoni*-infected individuals before (0) and 3, 6 and 12 months after treatment with PZQ. The bars indicate the median and interquartile ranges of 5 and 95% and the dotted line indicates the cut-off point (0.235)

A subgroup of 20 participants infected with *S. mansoni* was treated with PZQ and re-examined 3, 6 and 12 months after treatment. These participants were egg positive before treatment and negative during all follow-up fecal exams. Before treatment, the median absorbance readings was 0.200, increasing to 0.253 after 3 months and to 0.453 after 6 months, and decreasing to 0.349 after 12 months of treatment. However, there were no significant differences in the median OD readings between the different time points (Fig. 5).

## Discussion

Since the implementation of the Schistosomiasis Control Program in Brazil, in 1975, substantial advances in the control of the disease have been achieved. In addition, regular schistosomiasis control measures directly impacted the prevalence and intensity of *S. mansoni* infection, reducing mortality and the occurrence of severe forms of the disease (Brasil, 2014). Despite these significant advances, Brazil has still widespread endemic regions with active transmission, but with low positivity rates and reduced individual parasitic loads (Brasil, 2014). One of the factors that may be contribute to this situation is the use of parasitological techniques as the main form of laboratory diagnosis of intestinal schistosomiasis, either because of their high specificity or because of their effectiveness and relatively low costs (Speich *et al*., 2010; PAHO, 2014). However, these techniques have shown reduced or even poor performance, especially in individuals with low parasitic load (>100 epg) (Siqueira *et al*., 2016; Oliveira *et al*., 2018). As a consequence, these individuals are not treated and remain infected, and may contribute to the maintenance of residual transmission of the disease in endemic areas (Secor, 2014).

In this new scenario, it is essential to develop and apply more accurate diagnostic techniques that can contribute to the control and/or elimination of the disease (Rabello, 1997; Hawkins *et al*., 2016; Silva-Moraes *et al*., 2019*a*). Thus, any diagnostic technique that presents high levels of sensitivity and specificity is extremely important to provide the opportunity for treatment and, consequently, to promote the interruption of the transmission of the parasite in a certain area (Lustigman *et al*., 2012).

Techniques based on antibody detection have improved and complemented the diagnosis of schistosomiasis, especially in areas of low endemicity (Noya *et al*., 1992; Kinkel *et al*., 2012; Grenfell *et al*., 2013*a*, 2013*b*). Since parasite-specific antibody production is induced already during the first weeks after infection, antibodies can be detected even during pre-patency and before the onset of egg elimination in host feces (Lambertucci *et al*., 2013). On the contrary, a drawback of such serological assays is that they usually do not differentiate between active and past infections and might not even be used to indicate reinfection (Grenfell *et al*., 2014; Hinz *et al*., 2017; Silva-Moraes *et al*., 2019*a*). In addition, it is worth mentioning reports of antigenic cross-reactivity with other helminth infections (Tosswill and Ridley, 1986; Noya *et al*., 2001; Rodpai *et al*., 2021). In order to improve the performance of serological tests, several more specific antigens were selected, and recombinant antigens were used in ELISAs and evaluated for their diagnostic potential. The sensitivity of these assays varied considerably and attained 87% for SmE16, a protein expressed on parasite eggs (Moser *et al*., 1992); 77% for *S. mansoni* infections and 89.4% for *S. haematobium*, when the Sm22.3 antigen was used (Hancock *et al*., 1997); 55 and 60% with the use of Sm31 and Sm32 antigens, respectively, or increased to 75% when both antigens were combined (Klinkert *et al*., 1991).

In China, a group of researchers produced, in a prokaryote system, several multiepitope protein antigens of *S. japonicum* for the diagnosis of experimental schistosomiasis in goats and obtained a sensitivity and specificity of 97.8 and 100%, respectively (Lv *et al*., 2016), a much better performance than with the separately used antigen assays (Jin *et al*., 2010). These data reinforced the potential for the use of multiepitope antigens for improved diagnostic applications.

In this sense, a multiepitope antigen was constructed, expressed in *E. coli* bacteria, and used in an immunoassay for the diagnosis of intestinal schistosomiasis, in a group of selected Brazilian residents from localities with different endemicity profiles, e.g. endemic and non-endemic areas for intestinal schistosomiasis. During the first part of the statistical analyses of the ELISA IgG anti-SmME, the assay showed a reduced accuracy (61.8%) and only a weak agreement with the combined parasitological tests (*κ* = 0.26). This result should be analysed in 2 ways, related to the sensitivity and specificity of the test. The low sensitivity of the test may be related to the fact that not all the antibodies present in the individual serum samples from positive participants do recognize the very specific epitopes contained in the recombinant P1–P5 antigen. The human immune system can respond to an antigen with the production of antibodies against conformational and glycosylated epitopes (Rudd *et al*., 2001; Lisowska, 2002; Baum and Crocker, 2009; Petersen *et al*., 2009), which are not reproduced when recombinant proteins are produced in bacteria or from synthetic peptides, or when these are damaged by chemical substances during the purification process of the antigen (Fischer *et al.*, 1993; Misawa and Kumagai, 1999), thus, compromising the sensitivity of the serological test. Since the recombinant P1–P5 antigen were produced in a prokaryotic system, it does not contain the post-translational modifications carried out by eukaryotic cells (Marston, 1986), which may diminish or even prevent recognition by schistosome-specific antibodies in human sera. The expression of the recombinant antigen in other expression vectors, mainly eukaryotic ones, may increase the antigenicity of P1–P5 antigen and, consequently, improve the performance of the ELISA IgG anti-SmME. On the contrary, the performance of the test in correctly detecting egg-negative individuals may have been compromised by the underestimation of cases in supposedly egg-negative subgroups, especially those from endemic areas (NegE), due to possible false-negative results during the application of parasitological techniques for the reference standard (Oliveira *et al*., 2018). As already shown, in the current epidemiological scenario of intestinal schistosomiasis in Brazil, most infected individuals harbour a low parasitic load, which compromises the performance of parasitological techniques (Brasil, 2014). Furthermore, elevated levels of parasite-specific, circulating antibodies, in many cases due to past infections, may increase the number of false-positive results by serological tests (Grenfell *et al*., 2013*c*; Silva-Moraes *et al*., 2019*b*).

Most of the participants with intestinal schistosomiasis included in this study had a low parasitic load, i.e. less than 100 epg. This type of information is extremely relevant for the evaluation of any new test, since one of the most important limitations of the Kato–Katz technique is the detection of individuals with a low parasitic load (Enk *et al*., 2008; Siqueira *et al*., 2011; Oliveira *et al*., 2018). Thus, ELISA IgG anti-SmME performed well in the diagnosis of these individuals, with sensitivity ranging from 77.2 to 87.5%. On the contrary, the performance of the Kato–Katz technique, with the recommended reading of 2 slides from 1 fecal sample, was poor, especially in individuals with low or very low parasite counts and a detecting threshold of 12 epg per individual (Siqueira *et al*., 2016; Oliveira *et al*., 2018). In such a setting, the best performance of the Kato–Katz technique resulted by collecting 3 fecal samples and examining 2 slides from each of them, which achieved a maximum sensitivity of only 66.7% (Oliveira *et al*., 2018), which is considerably lower than the results obtained from our serological assay.

As for the kinetics of antigen-specific antibody production, the immunoreactivity of IgG antibodies against the recombinant P1–P5 antigen did not decrease, even after 1-year post-treatment. The presence of specific circulating antibodies can be explained by the persistence of some live worms in the hepatic portal system, without oviposition (Vendrame *et al*., 2001), by the release of several antigens after the death of the adult parasites (Roberts *et al*., 1987) and by the persistence of antigens in host tissues (Hassan *et al*., 1992). In this respect it was shown that the levels of parasite-specific antibodies can remain elevated for 2 years or more (Jones *et al*., 1992).

The selected serum samples included in our study belonged to residents from regions with very different epidemiological situations for schistosomiasis (endemicity from high to very low, or absent) and for other intestinal helminth infections. It is noteworthy that especially the egg-negative individuals were characterized by extended and thorough parasitological techniques to increase the reliability on their actual parasitological status. However, information on history of parasitic infections in these individuals was not available, a variable of extreme importance, especially in individuals from highly endemic schistosomiasis areas, who were partly included in this study. Considering that, to our knowledge, this is the first study that applied a multiepitope antigen for diagnosis of intestinal schistosomiasis. The ELISA IgG anti-SmME presented a good sensitivity in individuals with reduced parasitic loads, but further studies with increased numbers of schistosomiasis-infected individuals and individuals with intestinal helminth infections, as well as with endemic and non-endemic controls, should certainly be carried out to guarantee a more precise and empowered statistical evaluation of the assay. Also, an age-group-dependent evaluation of the assay might be of importance, with special attention to individuals with a low parasitic load.

In summary, we evaluated the performance of a *S. mansoni*-specific multiepitope antigen in an IgG ELISA with sera from resident individuals in endemic and non-endemic areas, and from individuals with geohelminth infections. The serological assay showed only fair concordance with the parasitological reference standard, composed of different parasitological methods, and a sensitivity and specificity of 81.1 and 46.1%, respectively. On the contrary, the detection of 87.5% of individuals with less than 100 epg and of 77.2% of individuals with 12 or even less epg, might turn this assay a valuable alternative for the detection of *S. mansoni*-infected individuals with low parasitic load. This should be evaluated in further studies with an increased number of individuals and separately for different age groups. Contrarily, the use of the assay to confirm the success of chemotherapy was not encouraged.

## Data Availability

Data previously obtained and used during the study are cited in the submitted article. The authors confirm that data supporting the findings of this study are available in the article and its supplemental materials.

## Supplementary material

For supplementary material accompanying this paper visit https://doi.org/10.1017/S0031182023000409. click here to view supplementary material
