# Supplementary figures and images for: Development and evaluation of an indirect ELISA using a multiepitope antigen for the diagnosis of intestinal schistosomiasis
Source: Parasitology. 2023 Apr 24;150(8):683–92. doi: 10.1017/S0031182023000409 (PMC10410369; doi:10.1017/S0031182023000409)

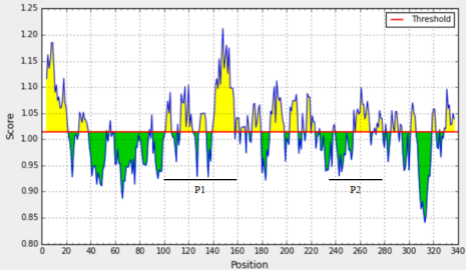

Supplement: Supplementary file 1 [file S0031182023000409sup.zip › S0031182023000409sup001.pdf]

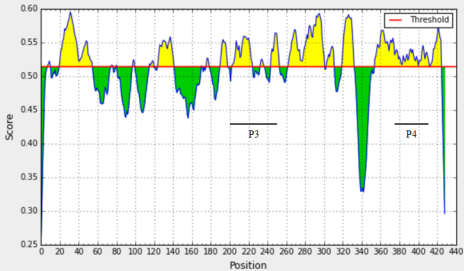

Supplement: Supplementary file 1 [file S0031182023000409sup.zip › S0031182023000409sup002.pdf]
